# Mediating effect of depressive symptoms on the relationship between digital literacy and cognitive function in older adults

**DOI:** 10.3389/fpsyt.2023.1248347

**Published:** 2023-09-21

**Authors:** Jae Woo Hong, You Jin Nam, Sunhwa Hong, Hyun Woong Roh

**Affiliations:** ^1^Department of Medicine, Gachon University School of Medicine, Incheon, Republic of Korea; ^2^Department of Psychiatry, Ajou University School of Medicine, Suwon, Republic of Korea

**Keywords:** cognitive function, depressive symptoms, digital literacy, mediating effect, older adults

## Abstract

**Introduction:**

Although several studies have examined the individual relationships among digital literacy, cognitive function, and depressive symptoms, few have integrated all three factors into a single model. This study aimed to address this gap by investigating the mediating effect of depressive symptoms on the relationship between digital literacy and cognition. In doing so, we hoped to contribute to a more comprehensive understanding of the complex interplay among these variables and their implications for mental health and well-being.

**Methods:**

Participants were 7,988 older adults (65 years or older) who participated in the Living Profiles of Older People Survey 2020. The main type of exposure was digital literacy (communication, information, media, and online transaction literacy). The main outcomes were depressive symptoms measured using the Short Geriatric Depression Scale of Korean version and cognitive function measured using the Mini-Mental State Examination score. Multiple linear regression and mediation analyses were also performed.

**Results:**

After adjusting for covariates, our analysis found a significant association between digital literacy and both depressive symptoms and cognitive function (β of four types of digital literacy and depressive symptoms = −0.123, −0.172, −0.702, and − 0.639, respectively; β of four types of digital literacy and cognitive function = 2.102, 2.217, 1.711, and 1.436, respectively). Moreover, our study showed that depressive symptoms play a mediating role in the relationship between media and online transaction literacy and cognitive function (95% CI of indirect effects = 0.0647–0.1212 and 0.0639–0.1277, respectively), implying an indirect pathway (digital literacy, depressive symptoms, and cognitive function).

**Discussion:**

This study sheds light on the relationship between digital literacy, depressive symptoms, and cognitive function in older adults. We found that depressive symptoms mediated the association between specific aspects of digital literacy (online transaction and media literacy) and cognitive function. Our results indicate that community-based digital literacy programs could be effective in reducing depression and preserving or improving cognitive function in older adults.

## 1. Introduction

The prevalence of dementia is projected to double every 20 years owing to an aging global population (1). Dementia is defined as a loss of memory, language, problem-solving skills, and other mental functions that impair a person’s ability to carry out daily tasks (2). Therefore, understanding the mechanistic pathways that lead to cognitive decline in older adults is a public health priority (3). Recent studies suggest that digital literacy is associated with cognitive functioning (4). Although extensive research has been directed toward the younger demographic (5, 6), there remains a paucity of studies centered on the elderly. It is pertinent to note that, for this older group, digital literacy might influence depression and cognitive function either positively or negatively. For example, digital literacy can potentially alleviate depression by fostering social connections and increasing access to mental health resources online (7, 8); in turn, reduced depression can enhance cognitive function by minimizing cognitive load and optimizing neural activity related to attention and memory (9, 10). The number of older adults who use digital devices, such as smartphones, has increased in many countries. In South Korea, among those aged 60 years or older, the percentage of smartphone users was 20% as of November 2012 and 76% as of July 2019 (11).

As the world population ages rapidly, depression has become a major public health concern among older adults (12). Depression affects not only the quality of life but also the physical and cognitive functioning of older adults (13). Recent studies suggest that the use of digital devices may serve as a protective factor against depression (14). The protective effects of digital literacy involve various possible psychological mechanisms, including increased neural plasticity and neurochemical modulation, as well as psychosocial mechanisms, including a sense of mastery and life satisfaction (15, 16).

Although depression is known to affect cognitive function, there is a strong likelihood that these three factors (digital literacy, cognitive function, and depressive symptoms) are closely connected (17, 18). Many studies have investigated the individual relationships among digital literacy, depression, and cognitive function in older adults (19). However, few studies have integrated all three factors into a single model, leaving a gap in our understanding of the complex interplay among these variables. Thus, the primary objective of this study was to evaluate the mediating effect of depressive symptoms on the relationship between digital literacy and cognitive function in a large sample of older adults in South Korea. By doing so, this study aimed to provide crucial insights into the potential factors contributing to mental health issues among older adults and inform targeted interventions to improve their digital literacy skills, cognitive function, and overall well-being.

## 2. Materials and methods

### 2.1. Participants

In the present study, 10,097 individuals aged 65 years or older who participated in the Living Profiles of Older People Survey (LPOPS) 2020 were selected. From September 14, 2020, to November 20, 2020, LPOPS 2020 constituted a comprehensive nationwide representative survey encompassing 17 cities and provinces across Korea. Participants who met the following criteria were excluded from the study: individuals with cognitive decline (*n* = 403), missing main outcome data [Mini-Mental State Examination (MMSE) score; *n* = 212], disabilities affecting survey performance (such as brain lesions, hearing impairment, or intellectual disability; *n* = 68), and missing covariates or main exposure data (*n* = 1,426).

**Figure 1:**
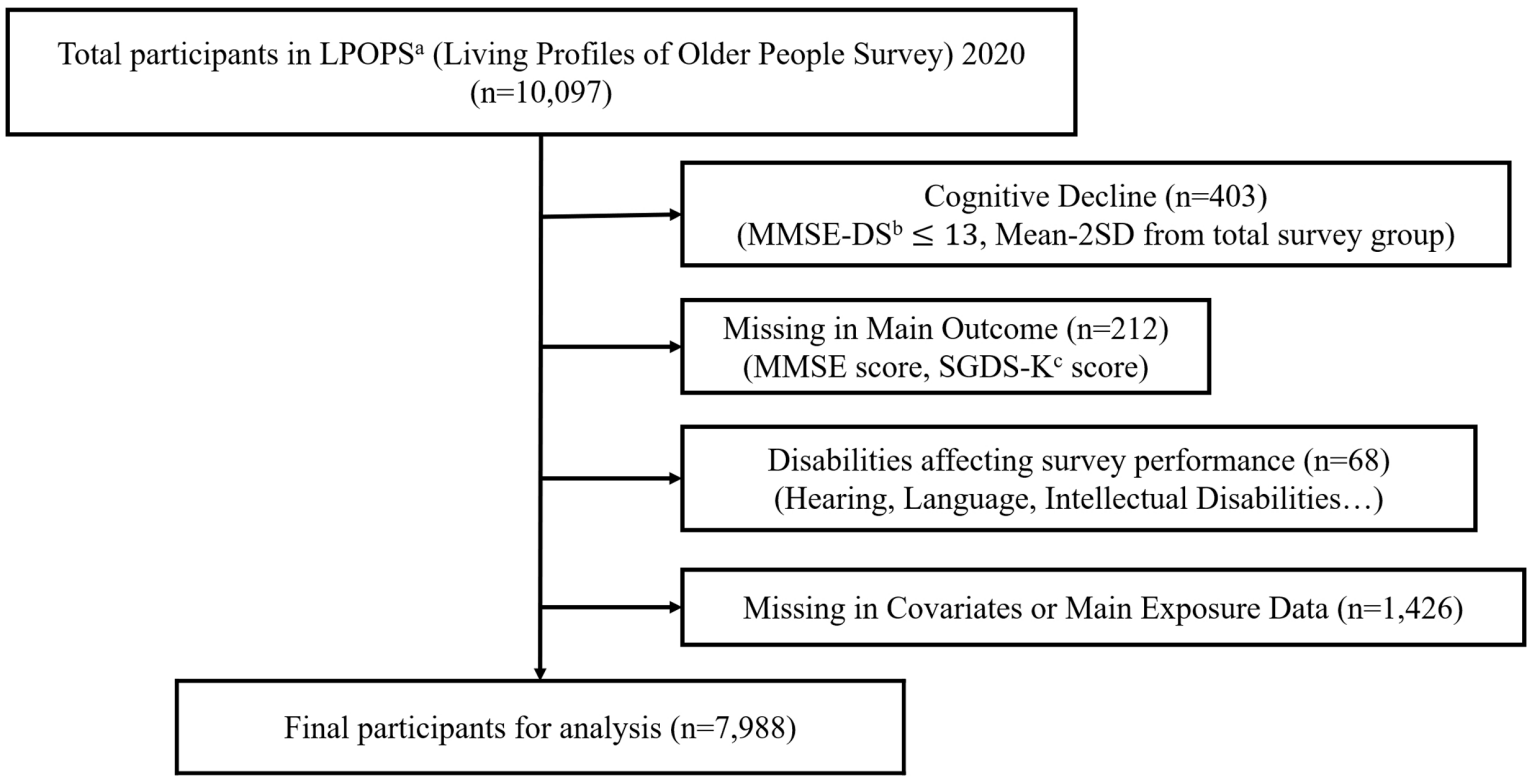
Flow chart of study participants. ^a^LPOPS, Living Profiles of Old People Survey. ^b^MMSE-DS, Mini-Mental State Examination—Dementia Screening. ^c^SGDS-K, Short Geriatric Depression Scale of Korean version.

For cognitive impairment, because the LPOPS 2020 is a community-based survey, this study calculated the mean and standard deviation from the survey population (*n* = 100, 97). The mean-2 SD of the survey was 13.7. Thus, participants with Mini-Mental State Examination-Dementia Screening (MMSE-DS) scores of 13 or below (*n* = 403) were excluded. A total of 7,988 participants were included in the final analysis. More detailed information is provided in Figure 1. The research received approval from Ajou University Hospital’s Institutional Review Board (AJOUIRB-EX-2023-317). During the survey, all participants provided their written consent to participate.

### 2.2. Assessment and measurements

#### 2.2.1. Digital literacy

Digital literacy has been defined as an umbrella framework for several complex and integrated literacies comprising skills, knowledge, ethics, and creative output in the digital network environment (20, 21).

Digital literacy includes “communication literacy,” “information literacy,” “media literacy,” and “online transaction literacy.” We defined four digital literacy sub-items using questions from the Living Profiles of Older People Survey (LPOPS) (22, 23).

The “communication literacy” domain is characterized by the capacity to send and receive messages using digital devices. Its assessment utilized the questions: “Can you receive messages using devices such as a PC, mobile phone, or tablet?” and “Can you send messages utilizing the same devices?” Those respondents who affirmed their proficiency in both these tasks were deemed “literate” in this domain, while those who were unable to complete one or both tasks were labeled “illiterate.”

The domain of “information literacy” pertains to the skill of searching for and retrieving information online through digital devices. It was gaged through the query: “Can you search for and view specific information (e.g., news, weather) using a PC, mobile phone, or tablet?” Participants who attested to their capability in this regard were labeled “literate,” while others were termed “illiterate.”

“Media literacy” encompasses the proficiency to engage with varied media content, such as listening to music or watching videos, via digital means. Assessment questions included: “Can you listen to music (e.g., MP3, radio) through devices like a PC, mobile phone, or tablet?” and “Can you watch content (e.g., movies, TV shows, YouTube) on these devices?” Respondents proficient in both activities were categorized as “literate” within this domain, while those lacking in one or both were considered “illiterate.”

Lastly, “online transaction literacy” is defined by the ability to execute reservations or financial transactions digitally. It was evaluated using the questions: “Can you use devices like a PC, mobile phone, or tablet for electronic transactions (e.g., online shopping, ticketing, reservations)?” and “Can you conduct financial operations (e.g., internet banking, securities) using these devices?” Respondents affirming their proficiency in both were deemed “literate,” whereas those unable to perform one or both tasks were categorized as “illiterate” in this domain.

#### 2.2.2. Depressive symptoms

Short Geriatric Depression Scale of Korean version (SGDS-K) was used to assess depressive symptoms (24). The SGDS-K comprises 15 binary questions that evaluate the presence of depressive and non-depressive symptoms. The scores on this scale range from 0 to 15, with higher scores indicating more severe geriatric depressive symptoms.

#### 2.2.3. Cognitive function

Cognitive function was evaluated using the MMSE-DS. It was developed to address the limitations of K-MMSE and provide an accurate dementia screening tool that reflects the cultural and demographic characteristics of the older adult population in Korea (25). The MMSE-DS includes items that assess orientation, memory registration and recollection, concentration, naming, language, understanding, and judgment, with scores ranging from 0 to 30; higher scores indicate higher cognitive function. This study used a cutoff score of 13 or lower to define cognitive impairment based on a community-based survey (i.e., the mean-2 SD of the total subjects). The test was administered according to standard guidelines developed during the development of the tool to ensure consistency in implementation. The reliability (Cronbach’s alpha = 0.83) and validity of the MMSE-DS have been previously established.

#### 2.2.4. Covariates

The covariates assessed in this study were age, sex, marital status, employment status, hobbies, years of education, current smoking, and the number of chronic diseases diagnosed by physicians. Years of education were divided into four groups: no education, 1–6 years, 7–12 years, and more than 13 years of education. A number of chronic diseases were defined as the number of chronic diseases diagnosed by a physician. These include cardiovascular, endocrine, musculoskeletal, pulmonary, neuropsychiatric, eye, ear, dermatologic, gastrointestinal, genitourinary, and other diseases.

### 2.3. Statistical analysis

The study utilized means and standard deviations for numerical variables and percentages and numbers for categorical variables. Depressive symptoms, measured using the total SGDS-K score, and cognitive function, measured using the total MMSE score, were continuous variables. A multiple linear regression analysis was conducted to examine the relationships among digital literacy, depressive symptoms, and cognitive function after adjusting for variables, such as age, sex, education level, marital status, and employment status. The analyses utilized Hayes’s (26) PROCESS plug-in program, which implements an ordinary least squares regression model and a bootstrap method to assess the statistical significance of the mediation and moderation effects. Specifically, the study utilized Model 4 to simultaneously investigate the relationships among digital literacy, cognitive function, and depressive symptoms, and 10,000 bootstrap samples were used to estimate the indirect effects of the mediating variables. Statistical significance was set at *p* < 0.05. All analyses were conducted using IBM SPSS v23.0 (SPSS Inc., Chicago, IL, United States).

## 3. Results

### 3.1. General characteristics of participants

The general characteristics of the study participants are presented in Table 1. The mean age of the participants was 73.4 years (Standard Deviation, SD = 6.1); 67.1% were male, and 32.9% were female. The mean number of hours spent using digital devices was 1.24 (SD = 1.2). The mean SGDS-K was 3.1 (SD = 3.2). The mean MMSE score was 25.3 (SD = 3.9).

**Table 1:** General characteristics of study participants.

Variables	All participants (*n* = 7,988)
Age	73.4 ± 6.1
Sex	
Men	5,361 (67.1)
Women	2,627 (32.9)
Quartiles of years of education	
No education	514 (6.4)
1–6 years	2,401 (30.1)
7–12 years	4,426 (55.4)
≥13 years	647 (8.1)
Marriage status	
Single or never married	31 (0.4)
Married or cohabiting	4,939 (61.8)
Widowed	2,690 (33.7)
Divorced	279 (3.5)
Separated	49 (0.6)
Employment status	
Employed	3,389 (42.4)
Unemployed	4,599 (57.6)
Alcohol consumption	
Ex or none smoker	7,069 (88.5)
Current smoker	919 (11.5)
Number of chronic diseases	1.8 ± 1.4
SGDS-K score	3.1 ± 3.2
MMSE score	25.3 ± 3.9

Values are represented as mean ± SD or *n* (%).

MMSE, Mini-Mental State Examination; SGDS-K, Short Geriatric Depression Scale of Korean version.

### 3.2. Classification of participants according to types of digital literacy

This study assessed participants’ digital literacy in four areas: communication, information, media, and online transaction. The distribution of participants across each domain is displayed in Table 2 as numbers and percentages. Among the participants, 70.0% were illiterate in communication, 62.8% were illiterate in information, 40% were illiterate in media, and 13.7% were illiterate in online transaction.

**Table 2:** Classification of participants by digital literacy.

Types of digital literacy	All participants (*n* = 7,988)
Communication literacy	
Illiterate	5,589 (70.0)
Literate	2,399 (30.0)
Information literacy	
Illiterate	5,013 (62.8)
Literate	2,975 (37.2)
Media literacy	
Illiterate	4,795 (60.0)
Literate	3,193 (40.0)
Online transaction literacy	
Illiterate	6,897 (86.3)
Literate	1,091 (13.7)

Values are represented as *n* (%).

### 3.3. Association of digital literacy with depressive symptoms

To evaluate the robustness of the correlations and their effect sizes while controlling for possible covariates, we conducted a multiple linear regression analysis. Compared to illiterate individuals, those who were literate in media and online transaction had fewer depressive symptoms (β = −0.702, 95% CI = −0.858 to −0.54, value of *p* < 0.001, and β = −0.639, 95% CI = −0.853 to −0.425, value of *p* < 0.001, respectively). More detailed information is provided in Table 3.

**Table 3:** Multiple linear regression analysis for association of digital literacy with depressive symptoms (*n* = 7,988).

Types of digital literacy	β	95% CI	*p* value
Communication literacy			
Illiterate (reference)			
Literate	−0.123	−0.297 to −0.067	0.155
Information literacy			
Illiterate (reference)			
Literate	−0.172	−0.327 to −0.017	0.03
Media literacy			
Illiterate (reference)			
Literate	−0.702	−0.858 to −0.547	<0.001
Online transaction literacy			
Illiterate (reference)			
Literate	−0.639	−0.853 to −0.425	<0.001

CI, confidence interval.

### 3.4. Association of digital literacy with cognitive functions

The subtypes of digital literacy were associated with varied levels of cognitive function. Among those with high communication literacy, interactive communication using digital devices was positively associated with cognitive function (β = 2.102, 95% CI = 1.906–2.298, value of *p* < 0.001). Similarly, individuals with high information literacy who searched for news using digital devices showed a positive association with cognitive function (β = 2.217, 95% CI = 2.038–2.396, value of *p* < 0.001), and those with high media literacy who listened to music and watched videos using digital devices also showed an association with higher cognitive function (β = 1.711, 95% CI = 1.532–1.890, value of *p* < 0.001). Furthermore, individuals with high online transaction literacy who engaged in online banking and reservations using digital devices had higher cognitive function (β = 1.436, 95% CI = 1.198–1.675, value of *p* < 0.001). More detailed information is provided in Table 4.

**Table 4:** Multiple linear regression analysis for association of digital literacy with cognitive function (*n* = 7,988).

Types of digital literacy	β	95% CI	*p* value
Communication literacy			
Illiterate (reference)			
Literate	2.102	1.906–2.298	<0.001
Information literacy			
Illiterate (reference)			
Literate	2.217	2.038–2.396	<0.001
Media literacy			
Illiterate (reference)			
Literate	1.711	1.532–1.890	<0.001
Online transaction literacy			
Illiterate (reference)			
Literate	1.436	1.198–1.675	<0.001

CI, confidence interval.

### 3.5. Mediating effect of depressive symptoms on the association between digital literacy and cognitive function

Our results revealed that depressive symptoms significantly mediated the association between several subcategories of digital literacy and cognitive function, thereby suggesting an indirect pathway (digital literacy ➔ depressive symptoms ➔ cognitive function). Media and online transaction literacy had an indirect effect on cognitive function mediated by depressive symptoms (indirect effect = 0.0916, 95% CI = 0.0647–0.1212; indirect effect = 0.0938, 95% CI = 0.0639–0.1277, respectively). More detailed information is provided in Table 5.

**Table 5:** Mediating effect of depressive symptoms on the association between digital literacy and cognitive function (*n* = 7,988).

Types of digital literacy	Direct effect	*p* value	Indirect effect	95% CI
Communication literacy				
Illiterate (reference)				
Literate	2.0571	<0.001	0.0039	−0.256 to 0.0335
Information literacy				
Illiterate (reference)				
Literate	2.1241	<0.001	0.0071	−0.186 to 0.0327
Media literacy				
Illiterate (reference)				
Literate	1.6184	<0.001	0.0916	0.0647–0.1212
Online transaction literacy				
Illiterate (reference)				
Literate	1.3346	<0.001	0.0938	0.0639–0.1277

CI, confidence interval.

## 4. Discussion

This study examined the interdependent associations among digital literacy, depressive symptoms, and cognitive function in older adults. We found that media literacy and online transaction literacy were associated with lower depressive symptoms in older adults, while communication literacy and information literacy were not significantly associated with lower depressive symptoms. Additionally, all four literacy types (communication, information, media, and online transaction literacy) were associated with higher cognitive function. These four literacies also demonstrated a direct effect on cognitive function. Furthermore, while the influence of digital literacy on depression and cognitive function can be complex, our analysis showed that media literacy and online transaction literacy indirectly affected cognitive function through a pathway mediated by depressive symptoms (digital literacy ➔ depressive symptoms ➔ cognitive function), whereas communication and information literacy did not exhibit this pattern.

Among the four types of digital literacy, communication and information literacy were not significantly associated with fewer depressive symptoms, which differs from the results of previous research (27). This may be due to the limited scope of digital communication or the unspecified identity of the interlocutors, which could have resulted in negative interactions. The study also lacked specificity regarding the type of news the participants were exposed to in the assessment of information literacy, which could have affected the results (21). In contrast, media and online transaction literacy were associated with fewer depressive symptoms, which is consistent with a previous study (28). This could be due to the consumption of positive content and social connectedness provided by media literacy, as well as the self-efficacy and self-mastery associated with online transaction literacy (29).

All four types of digital literacy (communication, information, media, and online transaction literacy) were significantly associated with higher cognitive function in older adults. This finding is consistent with those of previous studies (30). A possible mechanism is that digital literacy enhances cognitive function and brain plasticity in older adults by activating brain regions related to executive function, memory, and language processing and by modulating the negative effects of aging on brain activation (31, 32).

This study highlights the mediating role of depressive symptoms in the association between digital literacy and cognitive function among older adults. Building on prior research linking depression with cognitive decline, we propose a potential mechanism: enhanced digital literacy might alleviate depressive symptoms by reducing feelings of isolation, which in turn could improve cognitive function. Furthermore, digital literacy has both an indirect influence, through depressive symptoms, and a direct influence on cognitive function. This observation supports the idea that depressive symptoms partially mediate the relationship, underscoring their pivotal role in the dynamic interplay among digital literacy, cognitive function, and these symptoms.

However, our analysis also revealed a statistically significant reverse pathway: cognitive function influences depressive symptoms, which then affect digital literacy, suggesting a bidirectional relationship. While the primary pathway aligns with previous studies and is of notable interest, the reverse also warrants attention. To delineate the precise dynamics between these variables, a longitudinal follow-up study is crucial. The results reinforce our primary hypothesis, but also underscore the importance of further exploration into the potential bidirectionality of these associations.

To our knowledge, this is the first study employing a mediation model to investigate this relationship. These findings can guide the creation of interventions like community-based digital literacy programs. Such programs aim not only to boost the digital literacy and cognitive health of older adults but also to showcase their effectiveness.

The limitation of this study is its cross-sectional design, which prevents the assessment or confirmation of causal relationships between digital literacy and brain activation. Additionally, this study measured depressive symptoms based on a self-reported survey, which may not reflect the actual severity or duration of the participant’s symptoms. Second, this study adopted a vague definition of “digital literacy,” which lacked details about the types of communication and information relevant for assessing communication and information literacy. Lastly, although we controlled for all available confounders in our analysis, some unmeasured confounders could not be accounted for owing to data limitations. Nonetheless, this study has several strengths. First, the participants were recruited from a relatively large nationwide longitudinal community-based survey in Korea. Second, in addition to controlling for established or potential demographic predictors of depression, we considered clinically relevant factors such as tobacco and alcohol use, comorbid conditions, and disabilities.

In conclusion, media and online transaction literacy were associated with fewer depressive symptoms in older adults. All four digital literacy types were associated with higher cognitive function, and depressive symptoms act as mediators between certain dimensions of digital literacy (e.g., online transaction and media literacy) and cognitive functions. These findings are valuable for the development of effective interventions or public campaigns aimed at reducing depression and cognitive impairment from a targeted community mental health perspective.

## Data availability statement

The raw data supporting the conclusions of this article will be made available by the authors, without undue reservation.

## Ethics statement

The studies involving humans were approved by Ajou University Hospital’s Institutional Review Board. The studies were conducted in accordance with the local legislation and institutional requirements. The participants provided their written informed consent to participate in this study.

## Author contributions

JH: conceptualization, writing—original draft, and formal analysis. YN and SH: writing—review, editing, methodology, funding acquisition, and supervision. HR: conceptualization, methodology, investigation, writing—original draft, and formal analysis. All authors contributed to the article and approved the submitted version.

## Funding

This work was supported by the Korea Health Technology R&D Project through the Korea Health Industry Development Institute (KHIDI), funded by the Ministry of Health & Welfare, Republic of Korea (grant number: HI22C0724 and HR22C173405).

## Conflict of interest

The authors declare that the research was conducted in the absence of any commercial or financial relationships that could be construed as a potential conflict of interest.

## Publisher’s note

All claims expressed in this article are solely those of the authors and do not necessarily represent those of their affiliated organizations, or those of the publisher, the editors and the reviewers. Any product that may be evaluated in this article, or claim that may be made by its manufacturer, is not guaranteed or endorsed by the publisher.

## Abbreviations

LPOPS: Living profiles of older people survey
SD: Standard deviation
MMSE: Mini-mental state examination
MMSE-DS: Mini-mental state examination-dementia screening
SGDS-K: Short geriatric depression scale of Korean version
